# Exploring the Properties of Niobium Oxide Films for Electron Transport Layers in Perovskite Solar Cells

**DOI:** 10.3389/fchem.2019.00050

**Published:** 2019-02-06

**Authors:** Silvia Leticia Fernandes, Luiz Gustavo Simão Albano, Lucas Jorge Affonço, José Humberto Dias da Silva, Elson Longo, Carlos Frederico de Oliveira Graeff

**Affiliations:** ^1^Department of Chemistry, Federal University of São Carlos (UFSCAR), São Carlos, Brazil; ^2^Department of Physics, School of Sciences, São Paulo State University (UNESP), Bauru, Brazil

**Keywords:** niobium oxide film, perovskite solar cell, electron transport layer, methyl ammonium lead iodide, reactive sputtering

## Abstract

In this work, niobium oxide films were deposited by reactive magnetron sputtering under different oxygen flow rate and applied as electron transport layer in perovskite solar cells. It was found that the deposition made using 3.5 sccm of oxygen flow resulted in films with better electrical properties which helped the extraction of the photogenerated electrons to the external circuit, improving the J_sc_ and consequently the device efficiency. In addition, by photoluminescence measurements, we found a better charge transfer from perovskite to TiO_2_/niobium oxide film deposited at 3.5 sccm of oxygen flow.

## Introduction

Perovskite solar cells (PSCs) have emerged as one of the most promising photovoltaic technologies due to its high efficiency, low-cost and the facility to produce thin and flexible devices (Green et al., 2014; Kim et al., 2014; Park, 2015). However, to become commercial, a major issue is stability (Zhao et al., 2015; Li et al., 2016; Asghar et al., 2017).

The origin of instabilities in PSCs is associated with factors such as the organic components of hybrid perovskites and mobile ionic defects. Interfaces do also play a crucial role on the stability of the devices. At the electron transport layer (ETL)-perovskite interface, non-efficient interfacial charge extraction results in degradation of the perovskite material due to the photogenerated electrons that react with molecular oxygen resulting in superoxide (${\text{O}}_{2}^{-}$) species (Rajagopal et al., 2018). So, the choice of the ETL is crucial in order to have good electron injection and mobility preventing charge accumulation at the interfaces.

Many perovskite solar cells are constructed using titanium dioxide (TiO_2_) as ETL. Metallic oxides such as TiO_2_ have high resistivity and low electron mobility. Films that have higher conduction such as the organic [6,6]-Phenyl C61 butyric acid methyl ester (PCBM) produce good devices (Zheng et al., 2018), however, these materials have low stability.

Niobium pentoxide (Nb_2_O_5_) is a promising material to be used as ETL in perovskite solar cells due to its high stability. In our previous work, we found that the combination of compact Nb_2_O_5_ as hole blocking layer and TiO_2_ mesoporous produces more stable devices with less hysteresis (Fernandes et al., 2016; Gu et al., 2018). In addition, the band gap value of Nb_2_O_5_ could improve the V_oc_ of the cells (Kogo et al., 2015). However, as an oxide, the electron mobility in intrinsic Nb_2_O_5_ is low. To improve the conductivity of oxides ETLs doping is one possibility, however, it requires a fine control of the deposition parameters (Numata et al., 2018; Xiao et al., 2018). Changing the oxygen flow rate is a way to improve the conductivity without adding impurities to the system.

In this work we have systematically changed the oxygen flow under Nb_2_O_5_ deposition conditions and found that it is possible to increase the film conductivity by decreasing the oxygen flow rate. The decrease in oxygen flow rate induces oxygen vacancies which thus increases the film conductivity, leading to solar cells with better efficiency.

## Experimental Section

Fluorine doped tin oxide (SnO_2_:F) glass substrate (>7 Ω/sq sheet resistance) was purchase from Solaronix. The Nb target (99.9 %) was provided by Brazilian Metallurgy and Mining Company (CBMM). Lead (II) iodide (PbI_2_-99.998%) was purchased from Alfa Aesar. Spiro-MeOTAD (99%), bis(trifluoromethane)sulfonamide lithium salt (≥99.0%), 4-tert-butylpyridine (96%), ethanol, acetonitrile (anhydrous, 99.8%) and chlorobenzene (99.8%) from Sigma Aldrich. 2-propanol (max 0.005% H_2_O) and N-N dimethylformamide (DMF- max 0.003% H_2_O) from Merck. TiO_2_ paste (DSL 30NR-D), FK 209 Co(III) TFSL salt and methylammonium iodide (CH_3_NH_3_I) from Dyesol. All chemicals were used as received, without purification.

### Niobium Oxide Film Deposition

Niobium oxide films were deposited by reactive magnetron sputtering using a Nb target of 3″ in a Kurt J Lesker system. The deposition temperature was kept at ~500°C, the power at 240 W with argon flow rate at 40 sccm and the chamber pressure at 5.0 × 10^−3^ Torr. The oxygen flow rate was varied from 3 to 10 sccm and the deposition time was chosen in order to obtain 400 nm (to XRD and UV-Vis measurements) and 100 nm (for solar cell deposition as well other characterizations) thick films.

### Fabrication of Perovskite Solar Cells

Pscs were fabricated based on the mesoporous configuration: FTO/compact Nb_2_O_5_/mesoporous TiO_2_/ CH_3_NH_3_PbI_3_/ Spiro-OMeTAD/Au. Niobium oxide deposition was described in the previous section. The other layers were prepared following standard procedures (Burschka et al., 2013). First, the TiO_2_ mesoporous layer was deposited on top of the compact niobium oxide by spin-coating using a commercial paste from Dyesol diluted in anhydrous ethanol (150 mg/mL) at 4,000 rpm for 20 s and then the films were annealed at 500°C for 1 h. Then, a two-step deposition technique was used to synthesize the methylammonium lead iodide (MAPbI) perovskite films; two layers of 460 mg/mL PbI_2_ solution in anhydrous DMF was deposited at 6,000 rpm for 60 s, after each deposition, the films were annealed at 70°C for 10 min. A CH_3_NH_3_I solution (8 mg/mL in 2-propanol) was then dropped on PbI_2_, and left for 20 s. Just after the sample was spun at 4,000 rpm for 30 s and annealed at 100°C for 10 min. Spiro-OMeTAD was deposited on top of the perovskite film using a 72.3 mg/mL solution in chlorobenzene doped with 29 μL of FK209 cobalt complex (300 mg in 1 mL of acetonitrile), 18 μL of LiTFLS lithium solution (520 mg in 1 mL of acetonitrile) and 29 μL of 4-tert-butylpyridine. Finally, a film of 70 nm gold was thermally evaporated through a shadow mask defining a solar cell active area of about 0.34 cm^2^. The perovskite and spiro-OMeTAD layers were deposited inside of a glove box with nitrogen atmosphere and controlled H_2_O and O_2_, < 5 ppm.

### Characterizations

The morphological, structural and optical properties of the films were examined by X-ray diffraction (XRD), X-ray photoelectron spectroscopy (XPS), scanning electron microscopy (FE-SEM), Uv-Visible (UV-vis) and photoluminescence (PL) spectroscopy. XRD measurements were obtained using a Rigaku/RINT2000. UV-Vis was performed using a Varian Cary 50 UV-vis spectrophotometer and the optical band gap of the niobium oxide films was estimated from the optical absorption coefficients using Tauc's Plot. For XRD and UV-Vis measurements, 400 nm thicknesses films were deposited by controlling the time deposition. XPS was measured using ScientaOmicron ESCA+ with monochromatic X-ray source (Al Kα, hν = 1486.6 eV). A FEG-VP Zeiss Supra 35 model has used for the FE-SEM (high-resolution field emission scanning electron microscopy) images. PL measurements were performed with a HeCd laser model IK5451R-E, 442 nm. The spectrum was collected using an SR530 lock-in, a Thermo Jarrell Ash 27 cms monochromator and Hamamatsu R955 (500 V) photomultiplier. For PL measurements, FTO/niobium oxide/mesoporousTiO_2_/MAPbI configuration was used, and the samples were illuminated by the FTO side.

The current-voltage (I-V) curves as well as impedance spectroscopy of the oxide films were measured using PAIOS platform from Fluxim. The films were deposited on glass substrates and gold electrodes were evaporated resulting in a planar configuration; the distance between the electrodes was estimated in ~200 μm.

The PSCs characterization was performed using a Keithley 2400 source/measure unit in the dark and under simulated AM1.5G solar irradiation of 100 mW/cm^2^ from a calibrated solar simulator (Spectra-Nova).

## Results and Discussion

**Figure 1:**
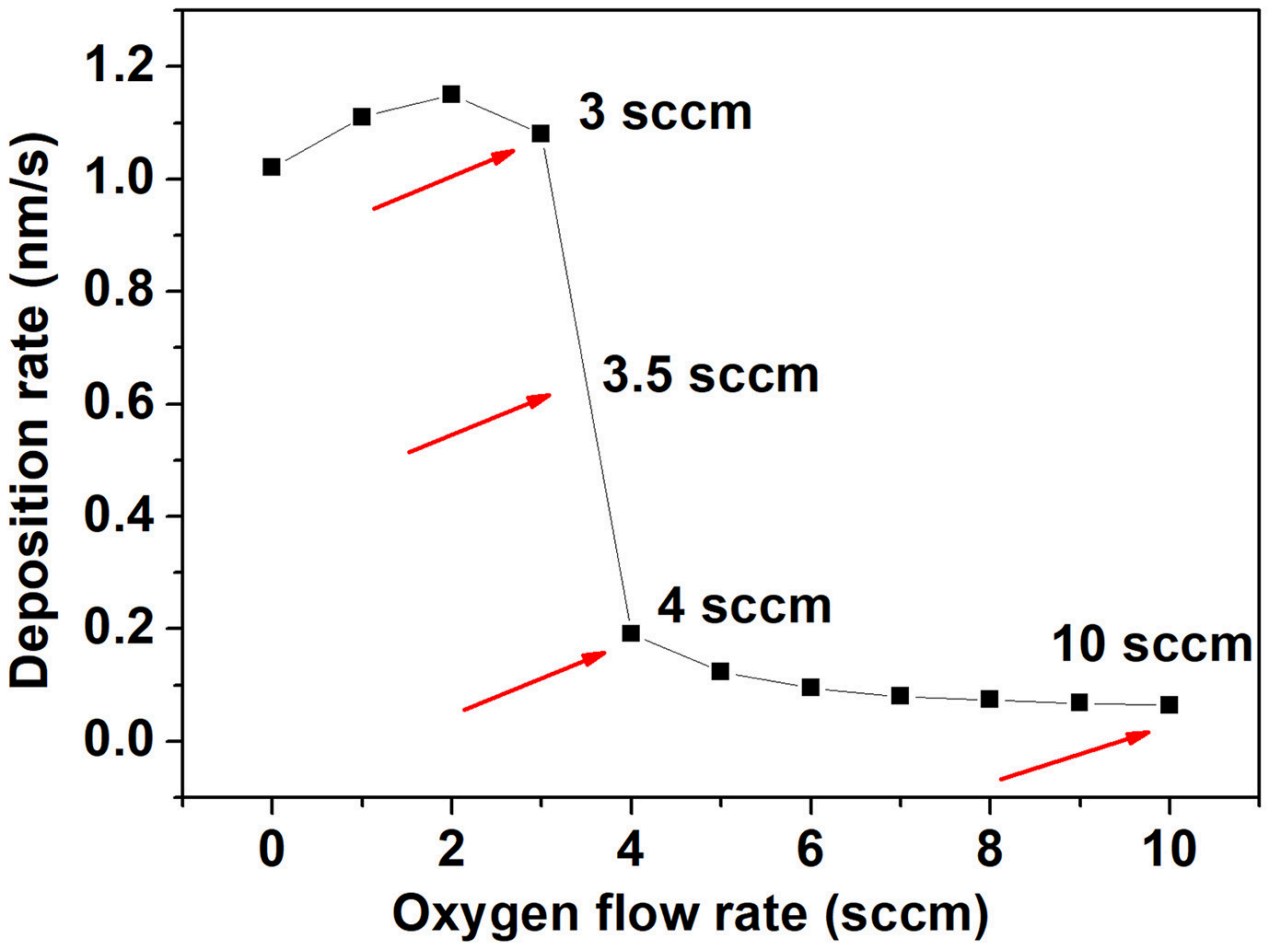
Deposition rate as a function of the oxygen flow rate used to deposit Nb_2_O_5_ films.

In a reactive sputtering deposition system there is a strong dependence of the deposition rate with parameters such as power or gas flow rate. We performed a study of the deposition rate as a function of oxygen flow rate and the results are shown in Figure 1.

As can be seen, for rates below 3 sccm the deposition rate is higher (1.1 nm/s). As the oxygen flow rate is increased, the deposition rate decreases. For 3 sccm the rate found is 1.1 nm/s, decreasing abruptly to 0.2 nm/s for 4 sccm. At oxygen flow rates higher than 4 sccm there is only a slight decrease in the deposition rate indicating that from 4 sccm and higher there is an excess of oxygen in the deposition chamber. Films deposited using 3, 3.5, 4, and 10 sccm are denoted 3NbO, 3.5NbO, 4NbO, and 10NbO, respectively.

**Figure 2:**
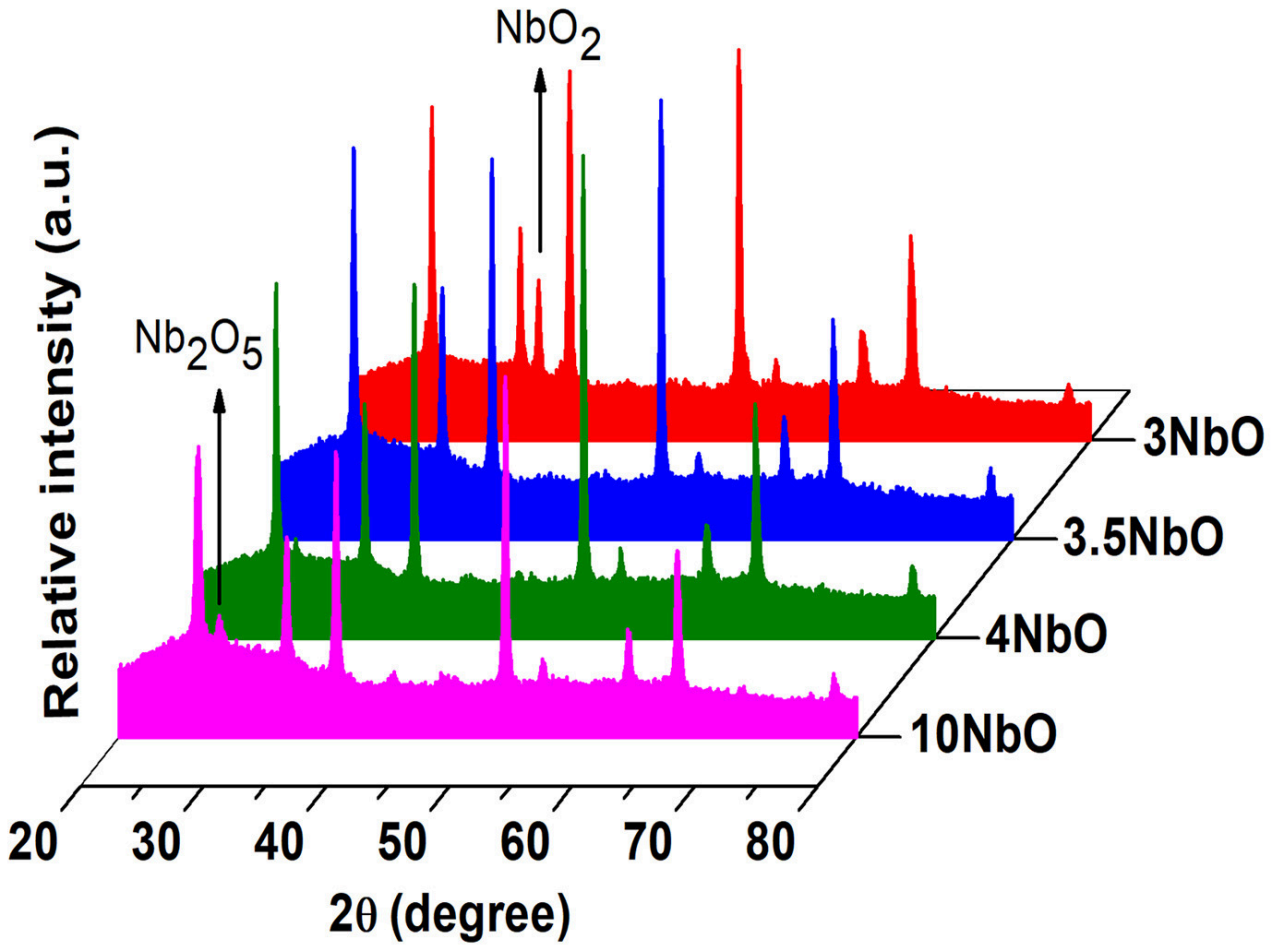
XRD spectra of the niobium oxide films deposited under different oxygens flow rates.

As previously mentioned, the deposition rate is strongly dependent on the oxygen flow rate. In addition, it can produce different niobium oxide phases such as NbO, NbO_2_, and Nb_2_O_5_, once niobium has three oxidation states: II, IV and V (Foroughi-Abari and Cadien, 2011; Rani et al., 2014; Al-Baradi et al., 2018; Lorenz et al., 2018). With reactive sputtering non-stoichiometric phases can also be produced such as Nb_22_O_54_ and Nb_12_O_29_ (Rani et al., 2014). From XRD, Figure 2, a phase transition between NbO_2_ and Nb_2_O_5_ is observed in the range of oxygen flow rates used. The most intense diffractions peaks are related to diffractions of FTO (substrate), at 35° one can observe a peak attributed to NbO_2_ for oxygen flow rates below 3 sccm. As the oxygen flow increases, the Nb_2_O_5_ peak located at 28.3° appears and becomes more noticeable in the 10NbO film. Thus, it can be concluded that oxygen flow rates higher than 3 sccm are needed to obtain the Nb_2_O_5_ phase. Therefore, NbO_2_ can be deposited with oxygen flow rates below 3 sccm.

**Figure 3:**
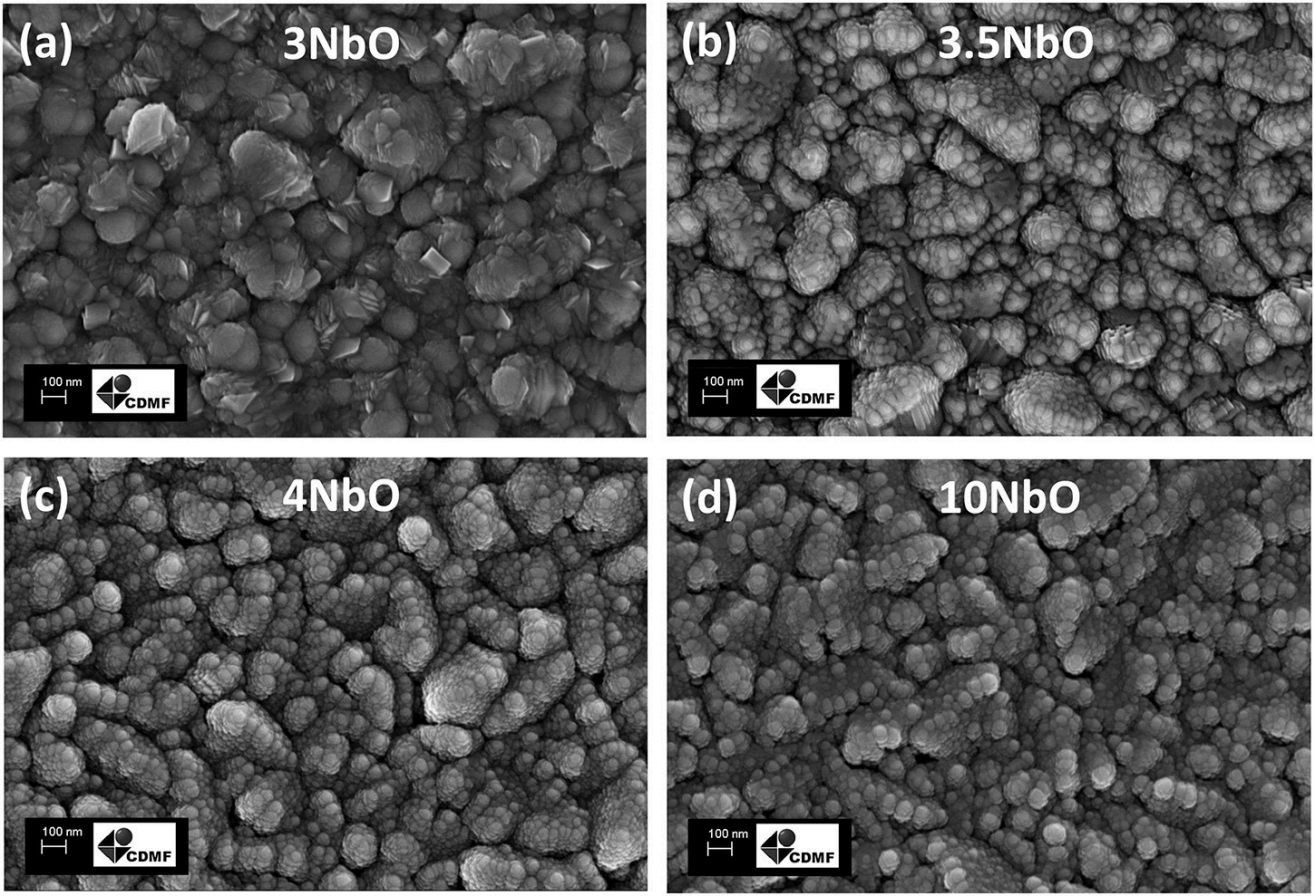
FE-SEM images of the 3NbO **(a)**, 3.5NbO **(b)**, 4NbO **(c)** and 10 NbO **(d)**.

The SEM images of the films are shown in Figures 3a–d. From the images it is possible to observe the similarity between the films, with nanometric spherical particles, apart from the 3NbO that showed sheets shape particles.

**Figure 4:**
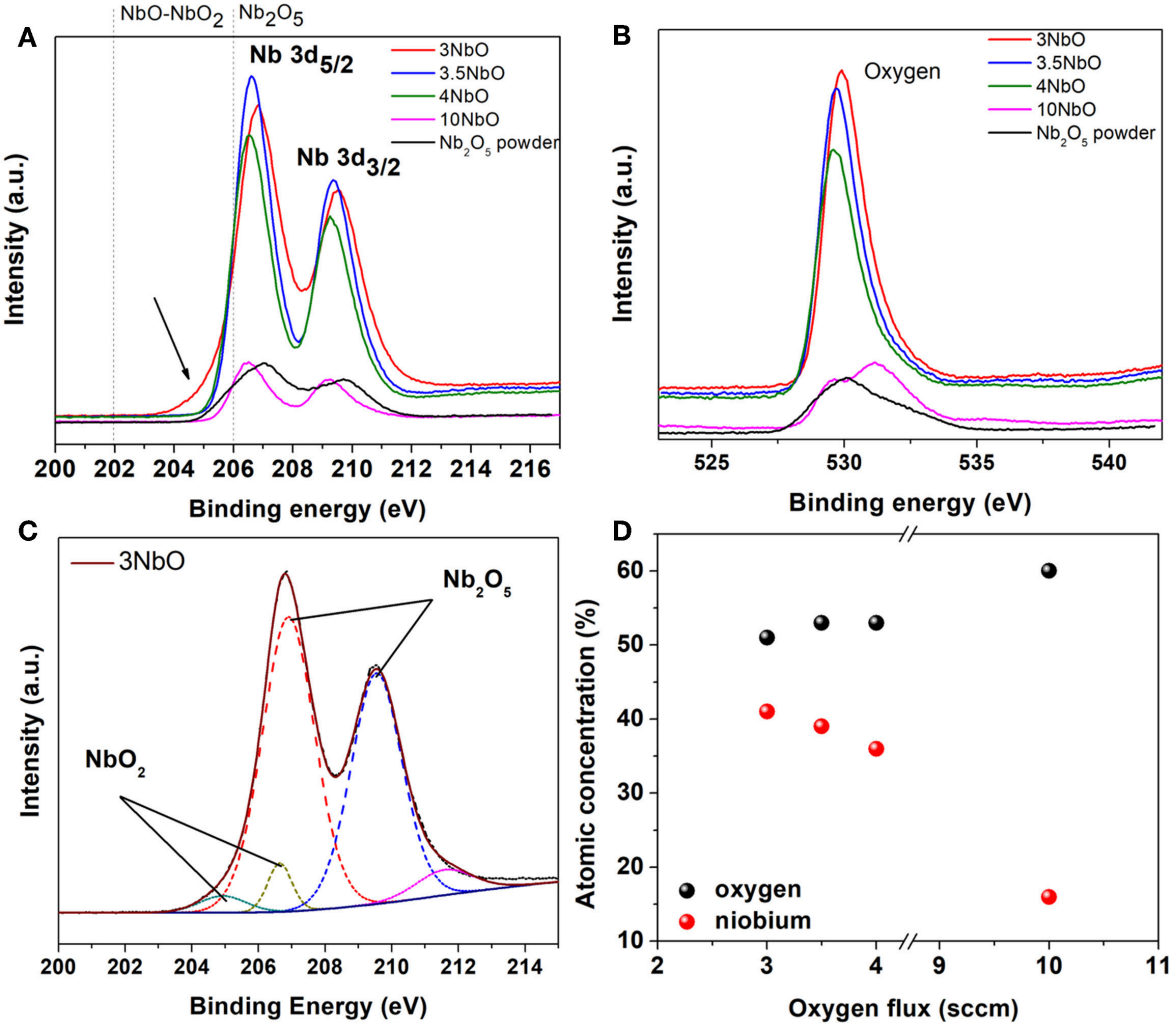
XPS spectra of the niobium oxide films at the Nb 3d edge **(A)**, O 1s edge **(B)**, the fitting curves (dot lines) of the 3NbO film **(C)** and atomic % of niobium and oxygen for different oxygen flow rates used to deposit the films **(D)**.

Figures 4A,B shows the Nb3d and O1s core level XPS spectra of the niobium oxide films. For all samples, two different components can be identified, characteristic of the Nb3d_5/2_ -Nb3d_3/2_ doublet, assigned to different bond states of niobium. The peak at ~207eV is related to the Nb^5+^ oxidation state, and thus to Nb_2_O_5_. A careful analysis of the 3NbO spectra (Figure 4A) reveals a broadening of the Nb3d_5/2_ peak. From the fit (Figure 4C) another component is found at a binding energy of 205eV, which is associated to Nb^4+^, thus related to NbO_2_. In fact, as observed in the XRD diffraction, in the 3NbO sample NbO_2_ is the dominant phase. The presence of the Nb_2_O_5_ phase in this sample is most probably due to the oxidation of the surface after exposure to air. Remembering that XPS compared to XRD is much more sensitive to the surface. Furthermore, there is no evidence of the Nb^4+^ peak for films deposited at oxygen flow rates higher than 3 sccm. From the XPS measurements, as expected, it can be observed the O/Nb ratio increasing as the oxygen flow rate increases, Figure 4D.

**Figure 5:**
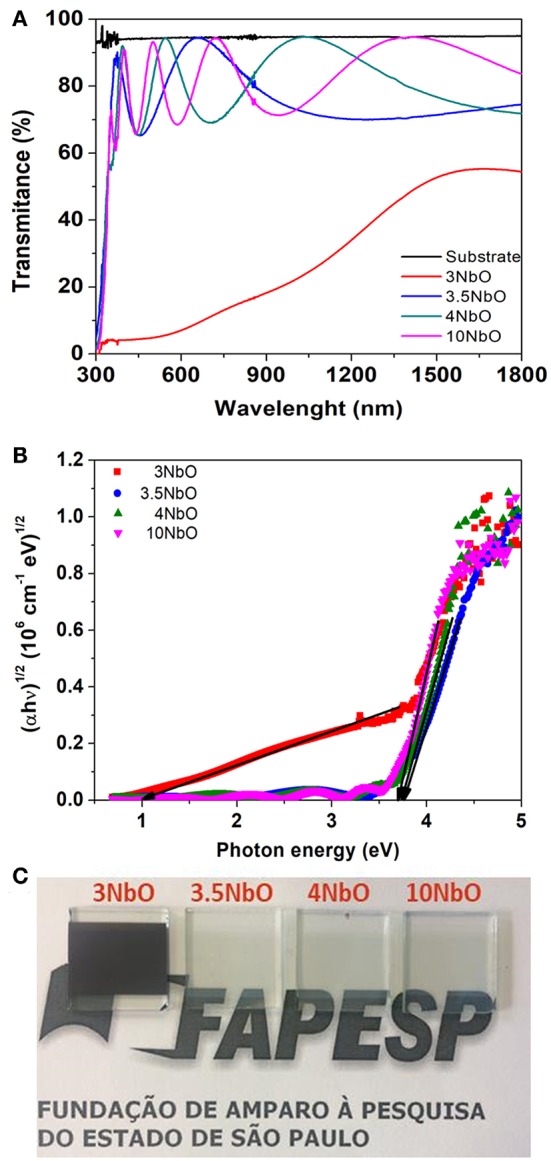
Transmittance spectra **(A)**, Tauc's plot **(B)** and picture of niobium oxide films **(C)**.

Figure 5A shows the transmittance spectra of the films. As expected, the transmittance of 3NbO film is much lower in contrast with the other films. Photographs of the films are shown in Figure 5C, where with the exception of 3NbO the others are transparent in the visible light range. The optical band gap energy of the films (Figure 5B) was calculated using Tauc's plot (Wood and Tauc, 1972). As expected, 3NbO shows a small band gap of ~1.1eV, characteristic of the NbO_2_ phase. A gap of about 3.77 eV was found for 3.5NbO, 3.72 eV for 4NbO, and 3.69 eV for 10NbO, indicating that in these films Nb_2_O_5_ is the dominant phase. Considering the films with larger gaps, there is a slight decrease in the optical band gap with oxygen incorporation. The different gaps may indicate the existence of intermediary energy levels between the valence and conduction bands (Pereira et al., 2018).

The presence of defects or even the variation of the stoichiometry has a direct influence on the conductivity of the material. It has been reported (Schäfer et al., 1969) that even a small change in the O/Nb ratio resulted in a significant conductivity change. The authors reported a variation from 3 × 10^−6^ to 3 × 10^3^ S/cm by changing the O/Nb ratios from 2.500 to 2.489. Note that these variations in the conductivity were not assigned to metastable phases or phase mixture, instead, to different concentrations of oxygen vacancies in Nb_2_O_5_ (Rani et al., 2014).

**Figure 6:**
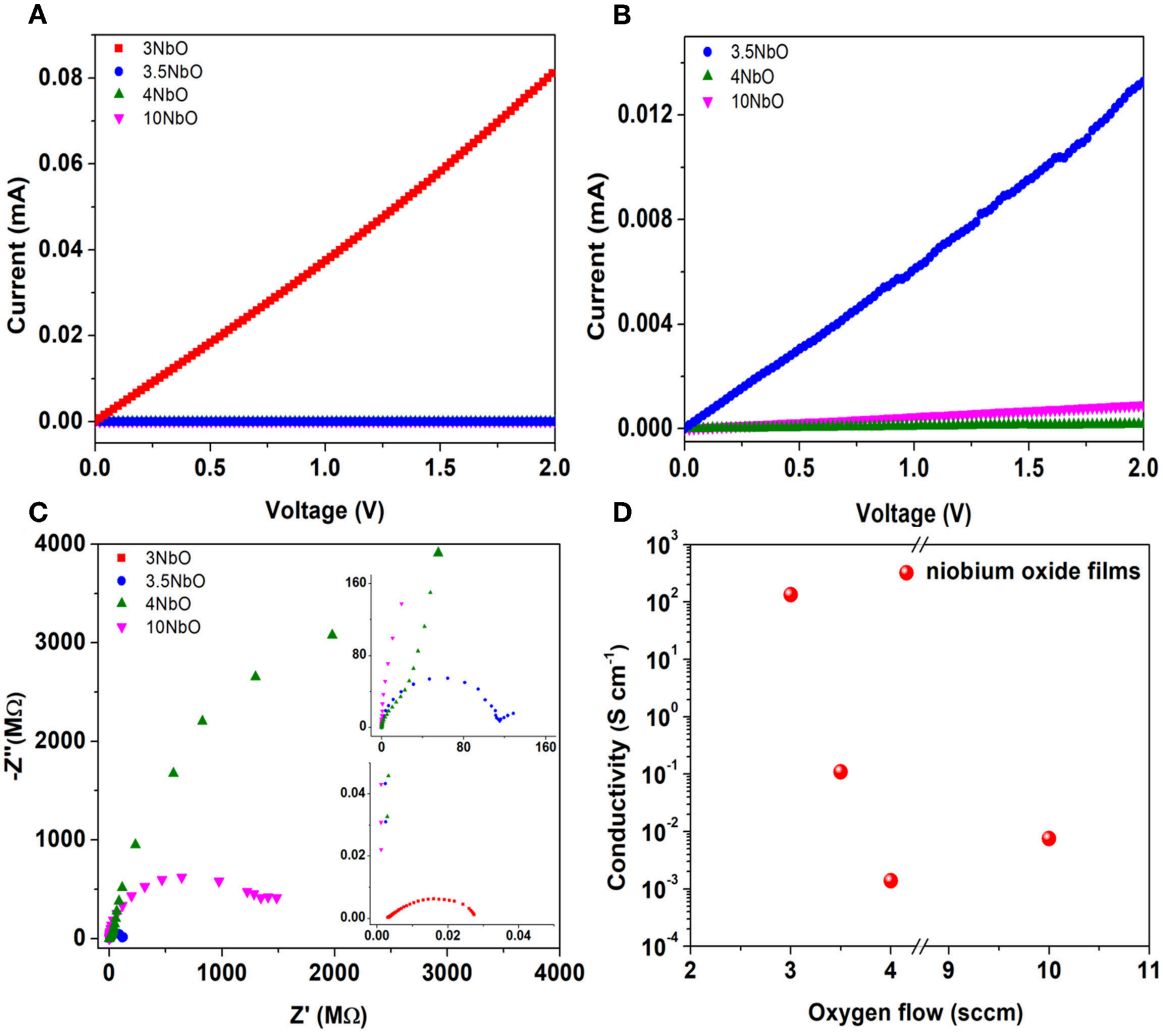
Current-voltage of niobium oxide films **(A)**, zoom of the 3.5NbO-4NbO and 10NbO curves **(B)**, Nyquist plot obtained from impedance spectroscopy **(C)** and calculated conductivity from current-voltage curves **(D)**.

Different conductivity values of the niobium oxide films were also observed as a function of oxygen flow rate during deposition, Figure 6. As can be seen in Figures 6A,B, the 3NbO has the highest current followed by 3.5NbO while 4NbO has the lowest. From the XRD, 3NbO has NbO_2_ as the main phase. Electrically, NbO_2_ is characterized as a semiconductor with high conductivity (Nico et al., 2016). On the other hand, Nb_2_O_5_ is typically a semiconductor with low conductivity. From 3.5 to 4 sccm the conductivity decreases as a result of a decrease in oxygen vacancies. As the oxygen flow increases the excess oxygen in the plasma (10 sccm) leads to a slight increase in the conductivity. This effect can be the result of the enhanced electron release from oxygen vacancies and/or oxygen desorption by generation of superoxide species, leading to a decrease in oxygen interstitials. Interstitial oxygen acts as a trap center for free carriers (Terheiden et al., 2014). It is important to notice that this trend is observed in samples deposited at different plasma powers,180 W, and equivalent oxygen flow rate (see Supplementary Materials S1, S2).

In Figure 6C, Nyquist plots obtained from impedance spectroscopy are presented. Considering the plot obtained for 3NbO, it is observed a semicircle shape typical from a parallel association of capacitor-resistor. This behavior is more associated with the current flowing through the resistor when the capacitor is fully charged, but in the present case, the resistance is low enough and the current can flow through the resistor before the capacitor be fully charged. This low resistance is associated with NbO_2_ phase. In the case of 3.5 NbO, when the main phase is Nb_2_O_5_, a semicircle shape is also observed followed by a low-frequency tail. This low-frequency tail could be associated with a capacitive charging process at the interface (Huggins, 2002; Wünsche et al., 2015). In 4NbO, the conductive is too small that only capacitive effects are present, no semicircle is observed because the high parallel resistance acts on the radius of the semicircle toward the very-low frequency region. The effect observed for 10 NbO is similar to 3.5 NbO, and it is associated with the slight increase in conductivity, as mentioned before. Furthermore, the conductivity was calculated and the values are shown in Figure 6D. The values were obtained using the relation between resistance (R), conductivity (σ), length (l) and cross-sectional area (A), R = l/σA. The resistance values were obtained through the angular coefficient of the curves obtained in Figures 6A,B.

**Figure 7:**
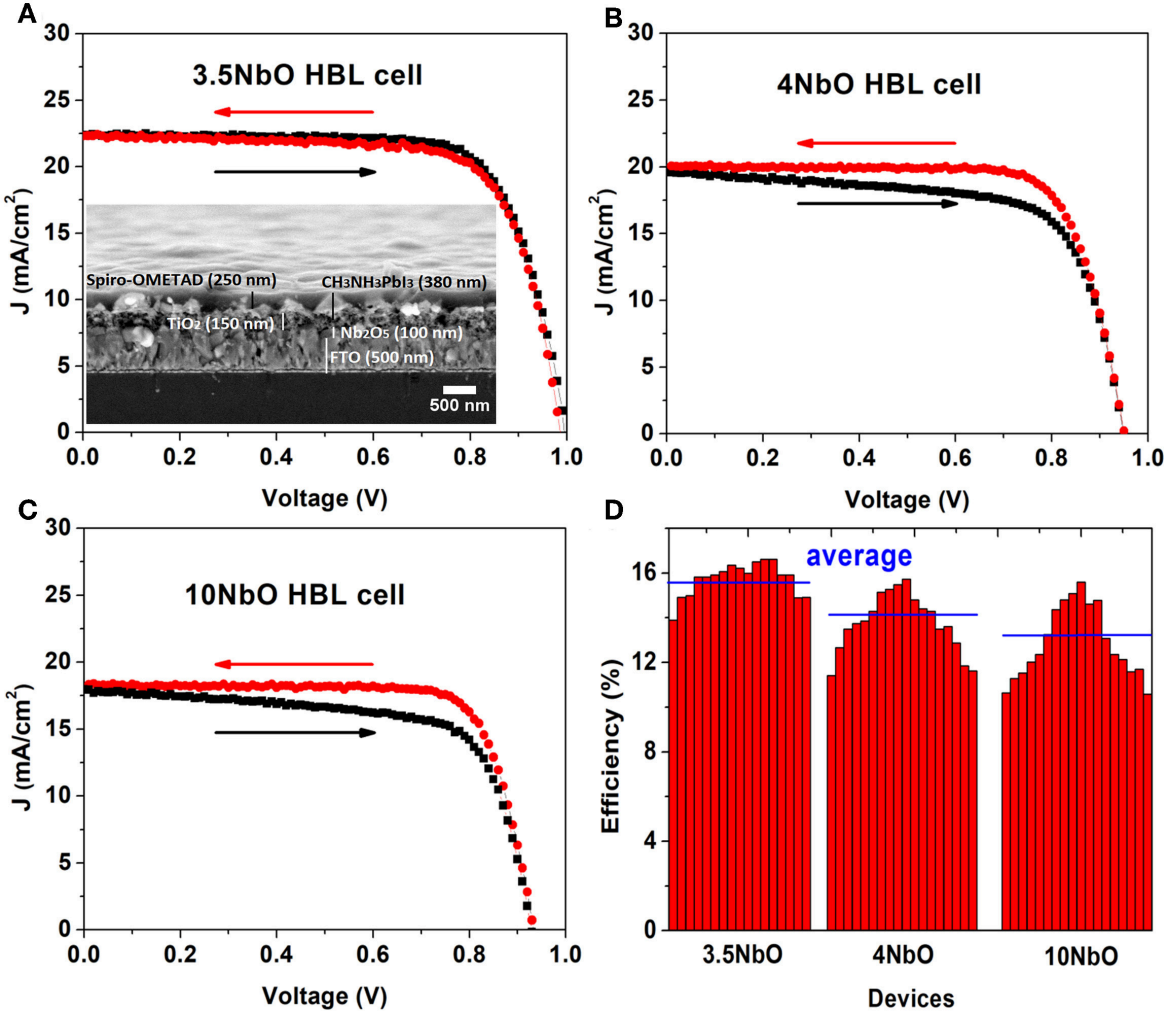
J-V curves of the perovskites solar cells using different niobium oxide ETLs **(A–C)** and average of the solar cells efficiencies **(D)**.

The niobium oxide films studied were used as ETL in perovskite solar cells. A cross section image of the device is shown inset on Figure 7A. For these solar cells, the 3NbO film was not used since transparency is an essential requirement in ETLs for n-i-p perovskite solar cells.

The J-V curves of the PSCs are presented in Figures 7A–C. Figure 7D shows the solar cell efficiency for different devices. The characteristic parameters summarized in Table 1. Each value presented in this table is the average of 8 different solar cells measurements and the standard deviations are shown in brackets.

**Table 1:** Solar cell parameters extracted from J to V measurements of the devices using different niobium oxide hole blocking layers.

**Average of 8 cells**	**Reverse**				**Forward**			
	**J_**sc**_ (mA/cm^**2**^)**	**V_**oc**_ (V)**	**FF (%)**	**Eff (%)**	**J_**sc**_ (mA/cm^**2**^)**	**V_**oc**_ (V)**	**FF (%)**	**Eff (%)**
3.5NbO cell	21.77 (1.50)	0.98 (0.02)	73 (4)	15.4 (0.85)	22.02 (1.72)	0.98 (0.01)	73 (1.00)	15.8 (1.35)
4NbO cell	20.20 (1.23)	0.96 (0.01)	68 (4.34)	13.2 (0.82)	20.71 (1.50)	0.96 (0.01)	72 (2.73)	14.4 (1.46)
10NbO cell	18.45 (1.79)	0.95 (0.02)	72 (3.33)	12.5 (1.69)	18.63 (1.90)	0.95 (0.01)	75 (2.65)	13.2 (1.61)

From the J-V measurements, there is a clear influence of the ETL on the performance of the cell. The cell made with 3.5NbO HBL has the best performance with the highest current.

The improved performance is attributed to the better conductivity of 3.5NbO ETL. As the resistivity of the niobium oxide films increases there is a decrease in the photocurrent which lowers the efficiency. From the point of maximum power of the J-V curves one can calculate the devices series resistance, this value is minimum for 3.5NbO devices, 290 Ω, and maximum, 410 Ω when 10NbO is used as expected.

As can be seen in Figure 7, the hysteresis of the J-V curve is also dependent on the oxygen flow rate. Hysteresis is the difference in the J-V curve of the forward (from 0 V to V_oc_) and reverse (from V_oc_ to 0 V) measurements. The J-V hysteresis increases from 3.5 to 10NbO based cells as a response to the decrease in conductivity. In our previous work, we proposed that charge accumulation is responsible for the hysteresis (Fernandes et al., 2016). Here, we attribute the effect to the same mechanism. As the niobium oxide films become less conductive, less photogenerated charges are extracted, accumulating at the interface of HBL-perovskite, thus these charges are responsible for the hysteresis observed in 10 and 4NbO HBL based devices. Note also that in our previous work 50 nm niobium oxide films (equivalent to 10NbO) were necessary to produce good devices. Here, the better charge extraction by 3.5NbO based solar cells allowed devices without hysteresis even when using 100 nm films, which has the advantage of producing films with less pinholes (Fernandes et al., 2016).

**Figure 8:**
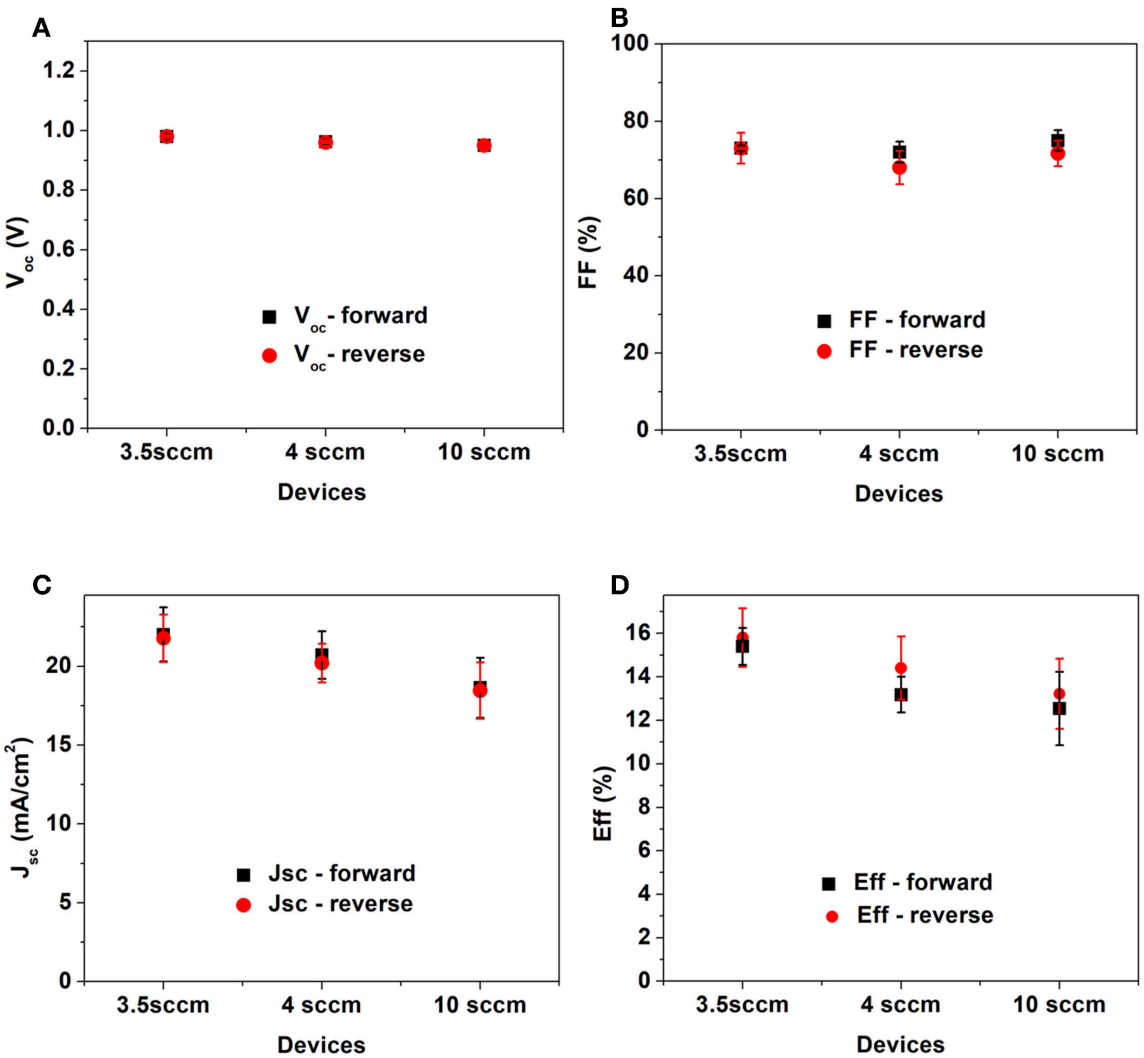
Parameters extracted from J-V curve: V_oc_, **(A)**, FF **(B)** J_sc_ **(C)** and efficiency **(D)**. The results are an average of 8 cells.

In Figure 8 the main solar cell parameters are displayed. It is possible to see that apart from V_oc_ other parameters are directly affected by the niobium oxide ETL. FF slight increases from 0.68 to 0.73, while the J_sc_ is mostly affected.

**Figure 9:**
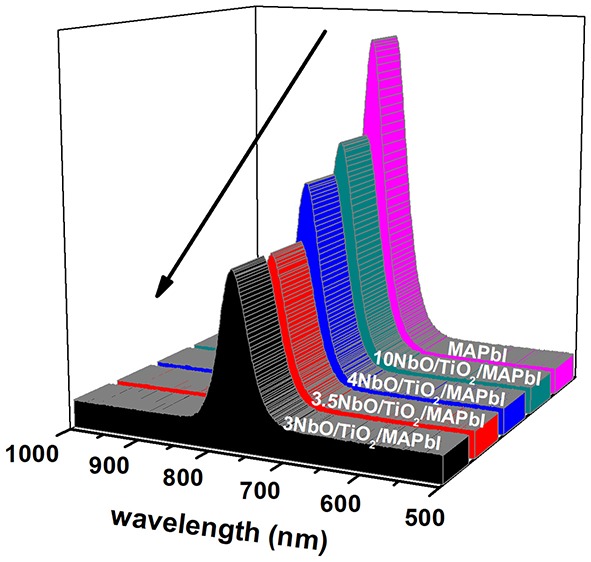
PL photoluminescence of niobium oxide/TiO_2_/MAPbI perovskite films.

The performance of the solar cell depends on charge transport and injection. PL results, Figure 9, show that the photoluminescence of MAPbI films decreases when the ETLs (mesoporous TiO_2_/niobium oxide) are put in contact with the perovskite. This result is a clear indication that the emission is suppressed by the charge transfer from MAPbI to ETLs. Electrons photogenerated in perovskite films are first transferred to TiO_2_ and then to niobium oxide film. As TiO_2_ is a mesoporous layer, the perovskite infiltrates and remais in contact with niobium oxide, so charges are also transfered directly from perovskite to niobium oxide. Our results show that PL emission is lowest when 3.5NbO/mesoporous TiO_2_ is used, indicanting that the charge transfer between these films and MAPbI is optimized.

## Conclusions

In this work, we have explored the influence of oxygen flow rate used to deposit niobium oxide ETLs and its influence on the performance of the devices. The oxygen flow rate strongly influences niobium oxide electrical conductivity. Considering the films used in the solar cells, 3.5NbO has the highest conductivity which produces solar cells with the highest efficiencies. As a consequence of the better electron extraction, 3.5NbO based devices has low hysteresis.

## Author Contributions

SF guided the role study. LGA helped with the data analyses and discussion of niobium oxide conductivity. LJA carried out the deposition rate measurements. JdS conducted the deposition of niobium oxide films. EL and CG helped with the discussion of all the experiments. All the authors contributed to writing and revising the manuscript.

### Conflict of Interest Statement

The authors declare that the research was conducted in the absence of any commercial or financial relationships that could be construed as a potential conflict of interest.
