# Supplementary material for: Exploring the Properties of Niobium Oxide Films for Electron Transport Layers in Perovskite Solar Cells
Source: Front Chem. 2019 Feb 6;7:50. doi: 10.3389/fchem.2019.00050 (PMC6372548; doi:10.3389/fchem.2019.00050)
Supplement: Supplementary file 1 [file Data_Sheet_1.docx]

Supplemental material for

**Exploring the properties of niobium oxide films for electron transport layers in perovskite solar cells**

**Silvia Leticia Fernandes^1*^, Luiz Gustavo Simão Albano^2^, Lucas Jorge Afonçço^2^, José Humberto Dias da Silva^2^, Elson Longo^1^, Carlos Frederico de Oliveira Graeff ^2^**

^1^ Chemistry Department, Federal University of São Carlos (Ufscar), São Carlos, São Paulo, Brazil

^2^ School of Science, São Paulo State University (Unesp), Bauru, São Paulo, Brazil


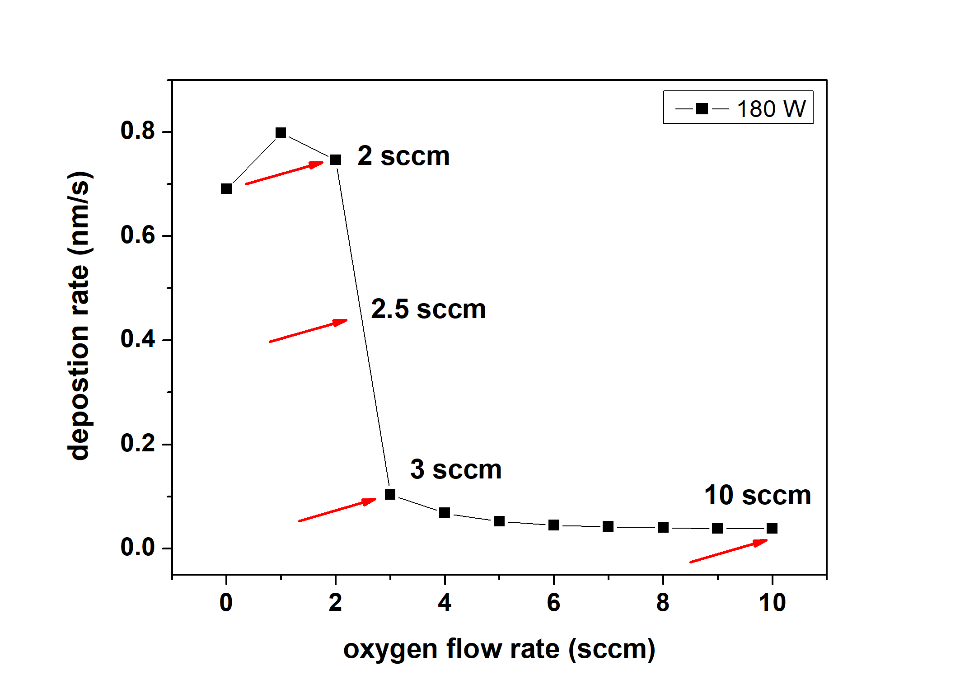


S.1: Deposition rate as a function of the oxygen flow rate used to deposit Nb_2_O_5_ at a deposition power of 180W.


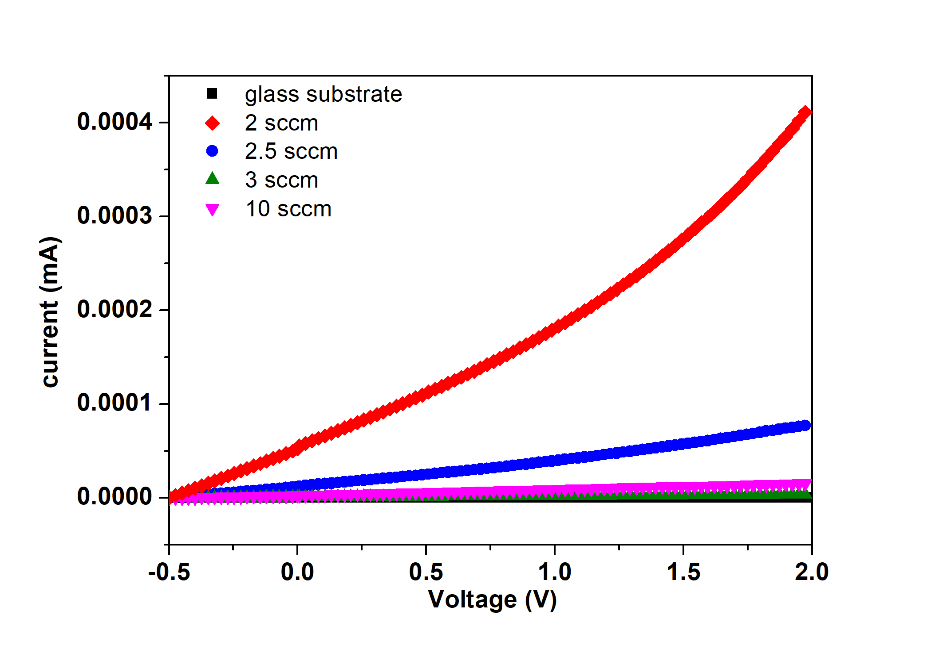


S.2: Current-voltage of niobium oxide films deposited at different oxygen flow rate and at a deposition power of 180W.
